# The topology of large Open Connectome networks for the human brain

**DOI:** 10.1038/srep27249

**Published:** 2016-06-07

**Authors:** Michael T. Gastner, Géza Ódor

**Affiliations:** 1 Yale-NUS College, 16 College Avenue West, #01-220 Singapore 138527; 2 MTA-EK-MFA, Research Center for Energy, Hungarian Academy of Sciences, P. O. Box 49, H-1525 Budapest, Hungary

## Abstract

The structural human connectome (i.e. the network of fiber connections in the brain) can be analyzed at ever finer spatial resolution thanks to advances in neuroimaging. Here we analyze several large data sets for the human brain network made available by the Open Connectome Project. We apply statistical model selection to characterize the degree distributions of graphs containing up to 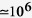 nodes and 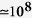 edges. A three-parameter generalized Weibull (also known as a stretched exponential) distribution is a good fit to most of the observed degree distributions. For almost all networks, simple power laws cannot fit the data, but in some cases there is statistical support for power laws with an exponential cutoff. We also calculate the topological (graph) dimension *D* and the small-world coefficient *σ* of these networks. While *σ* suggests a small-world topology, we found that *D* < 4 showing that long-distance connections provide only a small correction to the topology of the embedding three-dimensional space.

The neural network of the brain can be considered on structural (wired) as well as on functional connection levels. Precise structural maps exist only on very small scales. Functional networks, based on fMRI, are available on larger datasets. However a clear-cut relationship between them is largely unknown. Drawing parallels between neural and socio-technological networks, neuroscientists have hypothesized that in the brain we have small-world networks, both on a structural^1^ and functional level^2^. Small-world networks are at the same time highly clustered on a local scale, yet possess some long-distance connections that link different clusters of nodes together. This topology is efficient for signal processing^3^,^4^, but doubts have remained if the small-world assumption is generally true for the brain. Despite some evidence that functional networks obtained from spatially coarse-grained parcellations of the brain are small worlds^5^, at a structural cellular level the brain may be a large-world network after all^4^,^6^.

Like the small-world property, the hypothesis that functional brain networks have scale-free degree distributions became popular around the turn of the millennium^7^,^8^. The degree *k*_*i*_ of node *i* is defined as the number of edges adjacent to *i*. Because the degree is a basic measure of a node’s centrality, the probability Pr(*k*) that a node has degree *k* has played a key role in network science for a long time^9^. Especially physicists have popularized power law fits to observed degree distributions^10^,^11^. When such a fit is statistically justified, the network is called formally “scale-free”. Power laws play a crucial role in statistical physics, where they arise at transitions between an ordered and unordered phase because of the absence of a characteristic length scale. There are theoretical and empirical arguments that the brain operates near such a critical point^12^,^13^,^14^,^15^. For this reason it is plausible to assume that, on a functional level, the connectome’s degree distribution is also scale-free. More sophisticated statistical analyses of the functional connectome justify skepticism about the scale-free hypothesis^16^,^17^,^18^. Until now there are only few results for degree distributions of structural brain networks, and these do not show clear evidence for power laws^19^.

In this article we answer whether the structural connectome at an intermediate spatial resolution can be viewed as a scale-free, small-world network. We analyze large data sets collected by the Open Connectome project (OCP)^20^ that describe structural (rather than functional) brain connectivity. The particular data sets chosen by us were processed by members of the OCP from the raw diffusion tensor imaging data by Landman *et al*.^21^. Earlier studies of the structural network have analyzed much smaller data. For example the network obtained by Sporns *et al*., using diffusion imaging techniques^22^,^23^, consists of a highly coarse-grained mapping of anatomical connections in the human brain, comprising *N* = 998 brain areas and the fiber tract densities between them. The entire brain is made up of ~9 × 10^10^ neurons^24^, but current imaging techniques cannot resolve such microscopic detail. The networks investigated in this article have up to ~10^6^ nodes, which puts them on a scale that is halfway between the earlier coarse-grained view and the complete neural network.

One important measure which could not have been estimated previously because of too coarse-grained data is the topological (graph) dimension *D*. It is defined by


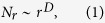


where *N*_*r*_ is the number of node pairs that are at a topological (also called “chemical”) distance *r* from each other (i.e. a signal must traverse at least *r* edges to travel from one node to the other). The topological dimension characterizes how quickly the whole network can be accessed from any of its nodes: the larger *D*, the more rapidly the number of *r*-th nearest neighbors expands as *r* increases. Different small-world networks can possess different *D*, for example due to their distinct clustering behavior^6^. Therefore, the level of small-worldness (quantified for example by the coefficient defined by Humphries and Gurney^25^) and the topological dimension contain different information.

Distinguishing between a finite and infinite topological dimension is particularly important theoretically. It has been conjectured that heterogeneities can cause strong rare-region effects and generic, slow dynamics in a so-called Griffiths phase^26^, provided *D* is finite^27^. Criticality^28^ or even a discontinuous phase transition is smeared over an extended parameter space. As a consequence, a signalling network can exhibit behavior akin to criticality, although it does not operate precisely on a unique critical point that sharply divides an ordered from a disordered phase. This phenomenon is pronounced for the Contact Process^29^, a common model for the spread of activity in a network. Subsequent studies found numerical evidence for Griffiths effects in more general spreading models in complex networks, although the scaling region shrinks and disappears in the thermodynamic limit if *D* → ∞^30^,^31^,^32^,^33^. Recently Griffiths phases were also reported in synthetic brain networks^34^,^35^,^36^ with large-world topologies and with modular organization, which enhances the capability to form localized rare-regions. Real connectomes have finite size, so they must possess finite *D*. If in real connectomes *D* remains small, these models hint at an alternative explanation why the brain appears to be in a critical state: instead of the self-tuning mechanisms that have been frequently postulated^37^,^38^, the brain may be in a Griffiths phase, where criticality exists without fine-tuning. Models with self-tuning require two competing timescales: a slow “energy” accumulation on the nodes and a fast redistribution by avalanches when the energy reaches the firing threshold. It is unclear if such a separation of timescales is realistic. Even if the brain were in a self-organized critical state with clearly separated timescales, Griffiths effects can play an important role due to the heterogeneous behavior of the system, frequently overlooked when modelling the brain.

## Open Connectome brain network data

The data sets analyzed in this article were generated by members of the OCP with the MIGRAINE method described by Roncal *et al*.^39^. In this section we will briefly summarize their methods. Afterwards we will describe our analysis which was based on the graphs publicly available from the OCP web site^20^. The raw input data used by the OCP consist of both diffusion and structural magnetic resonance imaging scans with a resolution of 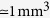 (i.e. the size of a single voxel). MIGRAINE combines various pieces of software into a “pipeline” to transform this input to a graph with 10^5^–10^6^ nodes.

As an intermediate step, the processing software first generates a small graph of 70 nodes^40^. For this purpose the image is downsampled into 70 regions taken from the Desikan gyral label atlas^41^. During this step the software also identifies the fibers in the brain with deterministic tractography using the Fiber Assignment by Continuous Tracking (FACT) algorithm^42^. As stopping thresholds a gradient direction of 70 degrees and a stopping intensity of 0.2 were used.

These fibers are then reanalyzed in the next step of data processing. The Magnetic Resonance One-Click Pipeline outlined by Mhembere *et al*.^43^ generates a big graph where each voxel corresponds to one node. First a “mask” is defined, for example the 70 regions included in the small graph. Then all data outside the mask are discarded and an edge is assigned to each remaining voxel pair that is connected by at least one fiber staying within the boundaries of the mask. This procedure will naturally produce hierarchical modular graphs with (at least) two quite different scales.

At this point each scan has been turned into a network with 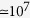 vertices and 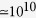 edges. However, due to the image processing algorithm (especially because the mask is chosen conservatively), many of these voxels will become disconnected and must be considered as noise. To clean up the data, all vertices outside the largest connected component are removed. According to Roncal *et al*.^39^ the remaining graph “keeps essentially all white matter voxels, consisting of ≈10^5^ vertices and ≈10^8^ edges”.

One important point to note is that two voxels *A* and *C* are linked by an edge even if there are other voxels *B*_1_, …, *B*_*n*_ between *A* and *C* on the same fiber. For example, if one traverses voxels *A, B, C* on a fiber, the edges (*A, B*), (*A, C*) and (*B, C*) are all part of the graph. Furthermore, the edges are undirected so that (*B, A*), (*C, A*) and (*C, B*) are also part of the graph because the FACT algorithm cannot provide information about the direction of an edge. Note that confounding factors such as the measurement technique, spatial sampling, measurement errors and the network-construction method can affect the graph data we downloaded^44^. We cannot control them, but tested the robustness of our conclusions by modifying one of the networks by neglecting a fraction of edges that might have arisen as a consequence of the transitivity rule. Additionally, we tested the effect of changing the reference null model from a nonspatial model (the ErdŐs-Rényi graph) to a spatial one (the random geometric graph, see section “Small-world coefficient” below).

To save space the OCP data files store only one of the directions (i.e. the upper triangle of the adjacency matrix) so that the opposite direction must be inferred from the data and inserted into the graph.

There were 3 different sets of big human brain graphs available from the OCP website^20^ with the abbreviations KKI (Kennedy Krieger Institute), MRN (Mind Research Network) and NKI (Nathan Kline Institute). The raw data are described by Landman *et al*.^21^, Jung *et al*.^45^ and Nooner *et al*.^46^, respectively. We analyzed the KKI graphs numbered 10 to 19 in more detail. Some graph invariants (e.g. degrees, clustering coefficients) were calculated and analyzed by Mhembere *et al*.^43^, but for the present study we have recalculated all invariants directly from the graph data available from the OCP website.

## Degree distribution

### Model selection

**Figure 1:**
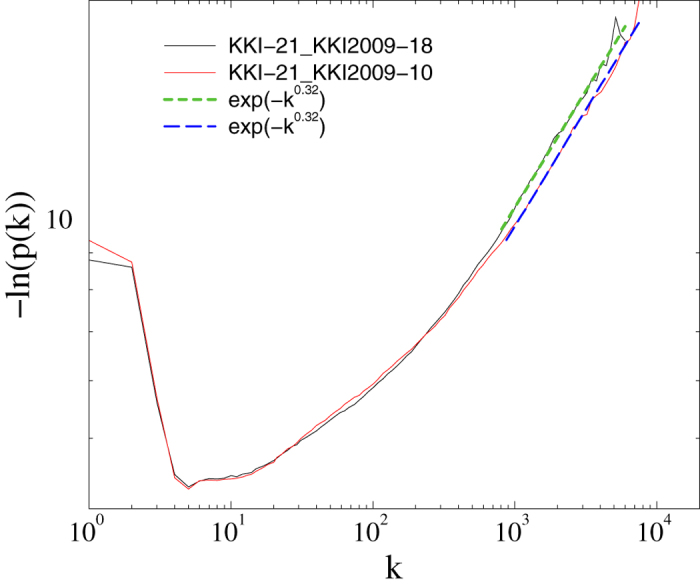
Empirical degree distributions of the KKI-10 and KKI-18 graphs (solid lines). Dashed lines show a rough-and-ready approach: ordinary least-squares fits for *k* > 1000 for the two graphs (short-dashed line for fit to KKI-18, long-dashed line for KKI-10) suggest stretched exponential tails. While ordinary least-squares fits are not a sound basis for model selection, we demonstrate in this article that there is indeed statistical evidence in favor of a generalized three-parameter Weibull distribution with a stretched exponential tail.

We want to assess how well different probability distributions fit the degrees of the OCP graphs. A first rough-and-ready visual attempt supports the hypothesis that the tails might be stretched exponentials, for example for the networks KKI-10 and KKI-18 in Fig. 1. However, such visual techniques have no inferential power. Even if the fitted parameters come from ordinary least-squares regression toolboxes, the fitted parameters in general do not converge to the true values even if the number of data points is large. Moreover, least-squares regression lacks a statistically principled criterion for comparing different models with each other.

A statistically sound framework for model selection is information theory. Here we adopt the information-theoretic methodology proposed by Handcock and Jones^47^ who fitted different functions to degree distributions in sexual contact networks. The key idea is that a good model should perform well in terms of two opposing objectives. On one hand the model should have enough flexibility so that it is able to fit the observed distribution. On the other hand it should have only a minimal number of parameters. In general, the more parameters we have at our disposal, the better we can fit the observation.

The goodness of fit can be quantified by the likelihood function which, under the assumption of independent observations, has the form


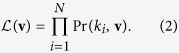


Here **v** is the set of parameters in the model, *N* the number of observations, and *k*_1_, …, *k*_*N*_ are the observed degrees. Alternatively we can write the likelihood as


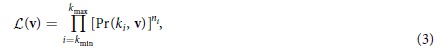


where *k*_min_ and *k*_max_ are the minimum and maximum observed degrees and *n*_*i*_ is the number of times we observe the degree *k*_*i*_. In the graphs KKI-10 through KKI-19, *k*_min_ is always equal to 1; *k*_max_ ranges from 5154 to 11241. In the extreme case of allowing as many parameters as there are observed degrees we can achieve a maximal likelihood of *e*^−*NH*^, where 
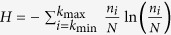
 is the Shannon entropy of the data. However, such a highly parameterized model is no longer informative, because it fits only the particular connectome used as input and sheds little light on general features that different connectomes might have in common.

Statisticians have proposed several “information criteria” to address this problem of over-fitting (e.g. Bayesian, deviance or Hannan-Quinn information criteria). These are objective functions that rate the quality of a model based on a combination of the likelihood 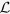 and the number of parameters *K*. In this study we apply the Akaike information criterion with a correction term for finite sample size^48^,


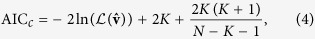


where 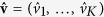 is the set of parameters that maximizes 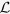; as before, *N* is the number of observations. The last term in Eq. 4 is a second-order bias correction which, although not in Akaike’s original formula^49^, gives more accurate results if 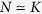
50.

While the absolute size of AIC_*c*_ is not interpretable, differences in AIC_*c*_ are informative and allow us to quantitatively compare different models^48^. If we denote the AIC_*c*_ value of model *j* by 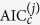 and the minimum over all models by 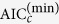, then the difference


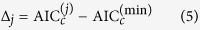


estimates the relative expected Kullback-Leibler distance between model *j* and the estimated best model^51^. As a rule of thumb, models with 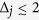 have substantial empirical support; models with Δ_*j*_ > 10 on the other hand are unlikely candidates to explain the data^48^.

Burnham and Anderson^48^ list many theoretical reasons in favor of model selection based on AIC_*c*_. The Bayesian information criterion (BIC), although almost equally popular, has been reported to yield poor results when used to fit power-law tails in probability distributions^52^ because it tends to underestimate the number of parameters. The Akaike information criterion penalizes less severely for additional parameters: in the limit 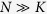 the penalty is asymptotically equal to the term 2*K* in Eq. 4, whereas the equivalent term in the BIC grows as *K* In(*N*). We carried out Monte Carlo simulations on synthetically generated probability distributions of the type described in the next section (see supplementary material). We found that model selection by AIC_*c*_ came close to the true number of parameters. Although the BIC showed acceptable performance, we confirmed that it indeed favors a too small number of parameters. We therefore advocate the use of AIC_*c*_ rather than BIC for fitting degree distributions.

### Candidate models

The first step of AIC_*c*_-based model selection is the definition of several candidate models that might generate the observed distribution. We denote as before by Pr(*k*) the probability that a node has degree *k*. The distinctive feature of different candidate models is the asymptotic decay of Pr(*k*) for 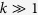. Only in this limit we can hope to find scale-free behavior if it indeed exists. Of course, all real networks are finite so that, strictly speaking, we cannot take the limit *k* → ∞. If we restrict ourselves to only a few high-degree nodes, we have too few data points for a meaningful fit. On the other hand, if we include too many low-degree nodes, then we may misjudge the correct asymptotic behavior of Pr(*k*).

We therefore assume for all candidate models that there is an optimal cutoff point *k*_*c*_ that separates nodes with degree ≤*k*_*c*_ from the region where a hypothesized asymptotic function *F (k*) can fit the data^47^,


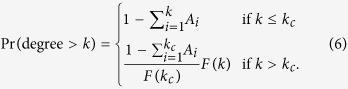


Each parameter *A*_*k*_ is chosen so that Pr (*k*) = *A*_*k*_ for *k* = 1, …, *k*_*c*_. Different families of candidate models can be defined by different functions *F* (*k*). We list all the functions *F* investigated in this study in Table 1. The exponential function (EXP) is the candidate that decays most rapidly in the right tail. The power law (POW) has two parameters: an exponent *β* > 0 and a constant *α* > 0 that shifts the function to the left or right. The tail 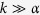 has the conventional power law form *F* (*k*) ∝ *k*^−*β*^. The discrete log-normal (LGN) and Weibull (WBL) distributions are represented by the usual distribution functions of their continuous namesakes. We also include two three-parameter models: a truncated power law (TPW) and the generalized Weibull distribution (GWB). In comparison to POW, TPW includes an additional exponential factor which is often used to mimic finite-size cutoffs in the right tail. GWB is a standard three-parameter generalization of WBL with an additional parameter *γ*, called location parameter, that shifts the distribution to the right or left^53^.

We can distinguish the members of each candidate model family by the choice of *k*_*c*_, the values of 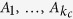 and *α, β, γ*. Model selection by AIC_*c*_ gives a natural criterion for the optimal parameters matching an observed distribution. It is a simple exercise to prove that 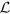 is maximized if *A*_*k*_ equals the observed relative frequency of nodes with degree *k*,


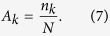


For a fixed value of *k*_*c*_, standard numerical algorithms can optimize the remaining parameters *α, β* and *γ* in Table 1 to maximize 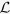. After calculating these maximum-likelihood estimators for every *k*_*c*_ between 0 and *k*_max_, we search for the value of *k*_*c*_ that minimizes AIC_*c*_ of Eq. 4. The number *K* of parameters that we have to insert into this equation is

- *K* = *k*_*c*_ + 1 for EXP,
- *K* = *k*_*c*_ + 2 for POW, LGN and WBL,
- *K* = *k*_*c*_ + 3 for TPW and GWB.

### Results of model selection

**Figure 2:**
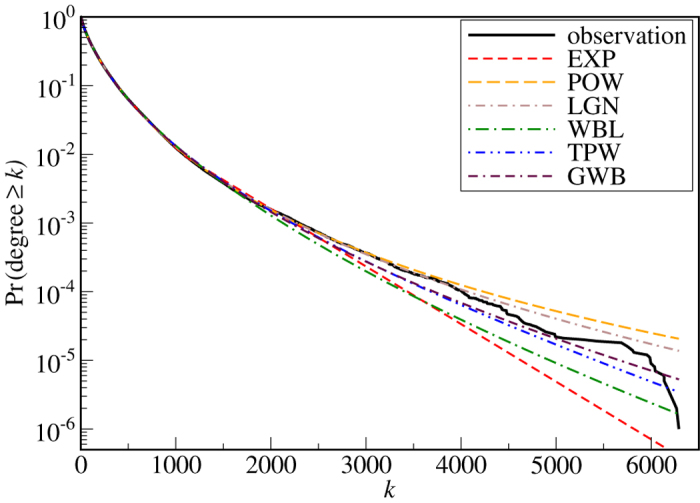
The maximum-likelihood distributions from each model family for matching the degree distribution of network KKI-18. In this example the generalized Weibull distribution is the best compromise in the right tail (see Table 2).

For each candidate model and each *k*_*c*_ = 0, …, *k*_max_ we compute the AIC_*c*_. We then determine the smallest 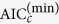 from the entire set and calculate for each model *j* the difference Δ_*j*_ defined in Eq. 5. In Fig. 2 we show for the example of the network KKI-18 the best-fitting distribution within each candidate model family. We have chosen a logarithmic scale for the ordinate to highlight the differences in the right tail. While the exponential and simple Weibull distributions decrease too rapidly, the power law and log-normal distributions decay too slowly. The truncated power law and generalized Weibull distributions are better in mimicking the overall shape of the distribution.

We find that, broadly speaking, this observation is typical of the ten investigated connectomes. As we see from Table 2, in 9 out of the 10 investigated networks we can achieve Δ_*j*_ < 10 with the GWB distribution. In 6 out of 10 cases, there is a similar level of evidence for the TPW model which has been previously hypothesized for functional brain networks^54^,^55^,^56^. For all other candidate distributions there is either none or very sporadic statistical support. When interpreting Table 2, one should bear in mind that even the best-fitting model is only “best” compared with the other tested candidates. True brain networks are of course far more complicated than any of our candidate models. Even if there were a “true” model, searching for “the” degree distribution of the human connectome is not a sensible endeavor because it would certainly be a highly complex model that is unlikely ever to be discovered and included in the set of candidates. As we discuss in the supplementary information, we can nevertheless sensibly ask which candidate model comes closest to the truth in the sense that it minimizes the Kullback-Leibler divergence. With this interpretation, we conclude that GWB is generally the best of our candidates. The fact that not all of the ten data sets are best fitted by the same model does not call this conclusion into question. Just as in traditional *p*-value based hypothesis testing, we also expect in AIC_*c*_-based model selection that the best general model is sometimes rejected by random chance for a concrete data sample.

A closer look at the fitted GWB values (Table 3) shows that, with the exception of KKI-16 where GWB is rejected by the AIC_*c*_, the *β* exponents lie within one order of magnitude suggesting a common trend if not even universality. This order of magnitude also agrees with the least-squares fit in Fig. 1.

## Dimension Measurements

**Figure 3:**
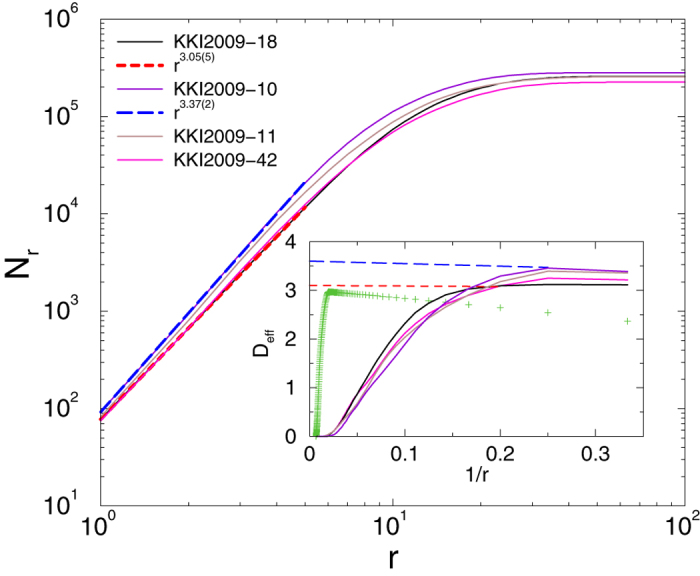
Number of nodes within graph distance *r* in the big KKI graphs. Dashed lines show power-law fits. Inset: local slopes defined in Eq. 8. Crosses correspond to measurements on a regular 100^3^ lattice.

To measure the dimension of the network^57^ we first computed the distances from a seed node to all other nodes by running the breadth-first search algorithm. Iterating over every possible seed, we counted the number of nodes *N*_*r*_ with graph distance *r* or less from the seeds and calculated the averages over the trials. As Fig. 3 shows, an initial power law crosses over to saturation due to the finite network sizes. We determined the dimension of the network, as defined by the scaling law (1), by attempting a power-law fit to the data *N*(*r*) for the initial ascent. This method resulted in dimensions between *D* = 3 and *D* = 4.

To see the corrections to scaling we determined the effective exponents of *D* as the discretized, logarithmic derivative of (1)


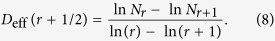


As the inset of Fig. 3 shows, *D*_eff_(*r*) tends to values below 4 even in the infinite size limit, but the narrow scaling region breaks down for *r* > 5. Furthermore, the extrapolated values for *D* of the connectomes exhibit an increasing tendency with *N* as we will now explain in detail.

**Figure 4:**
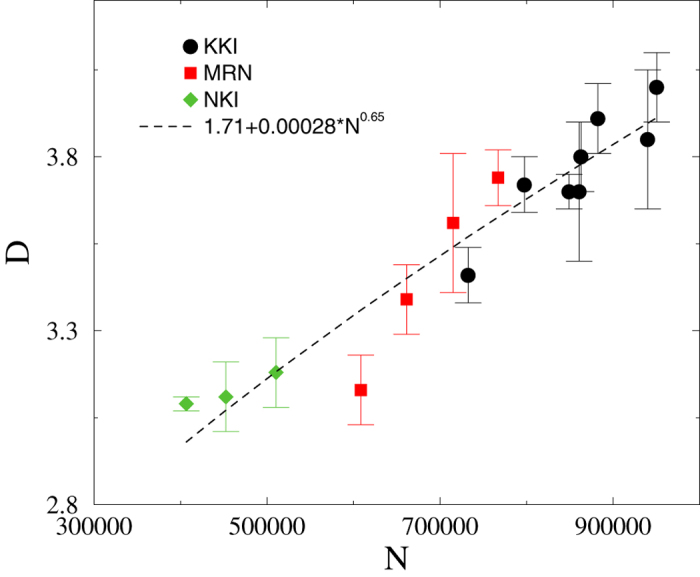
Topological dimension as a function of network size using different data sets. The line shows a power-law fit for the combined KKI, MRN, NKI results.

For better understanding we have also performed the analysis for other graphs besides KKI, possessing graph sizes in different ranges of *N*. The finite size scaling results are summarized in Fig. 4, where we also extrapolate to *N* → ∞. As one can see, the dimension values follow the same trend for KKI-, MRN- and NKI-graphs without any clear sign of saturation. A power-law fit to the data with the form *A* + *BN*^*C*^ is also shown, suggesting that *D* diverges for infinite *N*. It is tempting to extrapolate with this function to larger sizes or even to the infinite size limit. However, since we can rule out that the degree distributions are scale-free, we cannot assume that such extrapolated graphs faithfully represent connectomes of finer resolution. For example, using this power-law extrapolation we would overestimate their maximum degree *k*_max_.

Thus the present data does not permit claiming any particular numeric value for the dimension *D* of the true (i.e. microscopically resolved) brain connectome.

We cannot modify the algorithms that generate the OCP graphs, but tested the robustness by randomly removing 20% of the directed graph connections in case of the KKI-18 network. This makes the network partially directed, more similar to a real connectome. As a result the graph dimension did not change much: *D* = 3.2(2) instead of 3.05(1). Since the majority of edges are short, a random removal results in a relative enhancement of long connections, but *D* increases only slightly.

## Small-world coefficient

Small-worldness can be characterized by different definitions. One of them is the so-called small-world coefficient *σ*, which is defined as the normalized clustering coefficient (*C*/*C*_*r*_) divided by the normalized average shortest path length (*L*/*L*_*r*_),


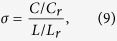


where the normalization divides the observed quantity (*C* or *L*) by the expectation value (*C*_*r*_ or *L*_*r*_) for an ErdŐs-Rényi (ER) random graph with the same number of nodes and edges^25^.

There are two different definitions of a clustering coefficient in the literature. The Watts-Strogatz clustering coefficient^58^ of a network of *N* nodes is


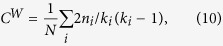


where *n*_*i*_ denotes the number of direct edges interconnecting the *k*_*i*_ nearest neighbors of node *i*. An alternative is the “global” clustering coefficient^59^, also called “fraction of transitive triplets” in the social networks literature^60^,


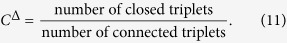


Both definitions are in common use, but values for *C*^*W*^ and *C*^Δ^ can differ substantially because Eq. 10 gives greater weight to low-degree nodes.

The average shortest path length is^61^


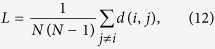


where *d (i, j*) is the graph distance between vertices *i* and *j. L* is only properly defined if the network is connected because otherwise the graph distance between some voxels is infinite.

**Figure 5:**
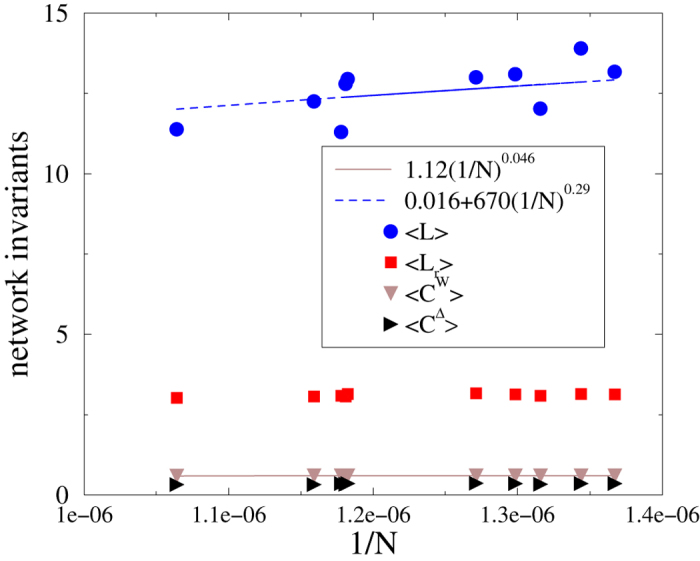
Network invariants as a function of 1/*N* for the investigated KKI graphs from Table 4. Lines show power-law fits.

We have calculated *C* and *L* for the largest connected components of several KKI networks directly from the edge lists on the OCP website^20^. The *C*^Δ^ values are about half of those for *C*^*W*^ (see Table 4). A finite size scaling analysis shows that the values of *L, C*^Δ^ and *C*^*W*^ decay by increasing the size *N* (see Fig. 5). For the average shortest path-length this decay is rather fast; a least-squares regression with the form *a* + *b*(1/*N*)^*c*^ results in 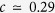 and 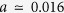. The constant is near zero within the precision of data, so in the infinite size limit we see small-world behavior. The clustering coefficients are almost constant; the power-law fit provides a very small slope: 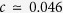 in agreement with the behavior of modular graphs^62^. The decreasing trends for *L, C*^*W*^ and *C*^Δ^ as functions of *N* are statistically significant at the 5% significance level; the *p*-values for *t*-tests of zero slope for the log-transformed data are 0.03, 0.002 and 0.04 respectively. Again, finite size scaling has to be interpreted with caution given that the topology is not scale-free.

As before, we tested the robustness, this time by deleting 10% randomly chosen undirected edges. We obtained very little changes: *L* = 11.37 (previously: 11.30), *C*^*W*^ = 0.538 (previously: 0.598) *C*^Δ^ = 0.322 (previously: 0.358).

Due to the transitivity of OCP graphs, one may question the validity of using ER graphs as a null model. Especially the high clustering can be at least partly attributed to the spatial embedding so that, for example, three-dimensional random geometric graphs^63^ are useful as comparison. Random geometric graphs have indeed a much higher clustering coefficient^64^, *C*^*W*^ = 15/32, a similar value to *C*^*W*^ in the OC graphs. To circumvent such problems Telesford *et al*.^65^ suggested another small-world criterion, using a “latticized” version of the graph for reference. However, as they pointed out, the latticization algorithm is computationally too demanding for large graphs. Their algorithm is in practice feasible only for up to 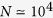 nodes, thus the necessary memory and run-times are prohibitive in our case.

We therefore kept the ER graphs as our null model and calculated the corresponding small-world coefficient *σ* in Eq. 9. We determined the clustering coefficient of the corresponding random networks *C*_*r*_ = 〈*k*〉/*N*, where 〈*k*〉 is the mean degree. We have computed the average path length of the corresponding ErdŐs-Rényi networks with the formula^66^


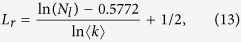


where *N*_*l*_ is the size of the largest component.

Applying these formulas to the KKI graphs, the last two columns of Table 4 show that *σ*^*W*^, 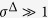 for all cases, suggesting small-world behavior according to this definition. These values do not show any tendency with respect to *N*: the *t*-tests for zero slope have *p*-values 0.45 and 0.72 for *σ*^*W*^ and *σ*^Δ^, respectively.

## Conclusions

Let us return to our introductory question: are the structural connectome graphs from the OCP database scale-free, small-world networks? As far as the adjective “scale-free” is concerned, the answer is clearly no. We have applied model selection based on the Akaike information criterion to 10 graphs comparing 6 different degree distribution models. The observed distributions are best fitted by the generalized Weibull function with a stretched exponential tail ∝exp (−*k*^*β*^). Most of the exponents *β* are between 0.2 and 0.5, which may hint at a universal trend. In some cases a truncated power law is a plausible alternative. However, the truncation occurs at a degree much smaller than the number of nodes in the network so that one cannot regard these distributions as scale-free.

Unlike the term “scale-free”, the adjective “small-world” does apply to the OCP connectomes in the sense that the small-world coefficients are much larger than 1. We have performed a finite size scaling analysis using several graphs and found no dependence between the number of nodes *N* and the small-world coefficients. The average path length, however, decreases as *N* increases. The resolution to this apparent paradox is that the average degree 〈*k*〉 increases with *N* so that there is an increasing number of shortcuts through the network. On the other hand, we obtained small topological dimensions characteristic of large-world networks. The dimensions show a tendency to grow as the sizes of the studied graphs increase. The limit *N* → ∞ here is taken by increasing *N* for a fixed voxel size. The absence of a scale-free degree distribution suggests that this limit may not be equivalent to fixing the brain volume and instead resolving the details of the connectome at an infinitely small scale. For this reason, it is difficult to judge whether Griffiths effects can be found in the brain, but the small topological dimensions that we have observed warrant further investigation.

Our analysis has been based on unweighted graphs. More realistically, however, the connectome is a weighted, modular network. Links between modules are known to be much weaker than the intra-module connections. Thus, future studies should take into account that signals in the brain propagate on a weighted, heterogeneous network, where generic slow dynamics is a distinct possibility^67^. The methods presented here to characterize degree distributions and topological dimensions can be generalized to the weighted case. We hope that, as more precise and finely resolved connectome data will become available, future research will be able to assess whether Griffiths phases can indeed occur in the brain.

## Additional Information

**How to cite this article**: Gastner, M. T. and Ódor, G. The topology of large Open Connectome networks for the human brain. *Sci. Rep.* **6**, 27249; doi: 10.1038/srep27249 (2016).

## Supplementary Material

Supplementary Information

## Figures and Tables

**Table 1:** Investigated candidate models for the degree distribution.

Model	*F*(*k*)
exponential (EXP)	*e*^−*αk*^
power law (POW)	*α*^*β*^ (*k* + *α*)^−*β*^
log-normal (LGN)	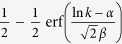
Weibull (WBL)	exp (−*αk*^*β*^)
truncated power law (TPW)	*α*^*β*^ (*k* + *α*)^−*β*^ *e*^−*γk*^
generalized Weibull (GWB)	exp [*α* (*γ*^*β*^ − (*k* + *γ*)^*β*^)]

**Table 2:** Smallest relative Akaike information criterion 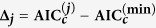 for each candidate degree distribution.

connectome KKI-…	Δ_*j*_					
	EXP	POW	LGN	WBL	TPW	GWB
10	1317.82	202.90	204.81	73.70	**0.00**	**3.52**
11	1245.42	17.39	**0.00**	38.75	**1.21**	**0.43**
12	992.62	43.28	21.83	174.19	**0.00**	**0.73**
13	767.95	155.43	72.68	221.50	**3.45**	**0.00**
14	792.10	117.26	124.21	85.74	**0.00**	**9.57**
15	1094.40	120.99	139.87	26.19	86.06	**0.00**
16	954.28	18.81	29.99	66.59	**0.00**	17.20
17	736.42	195.15	216.05	18.16	49.47	**0.00**
18	1168.50	109.51	109.90	185.59	51.81	**0.00**
19	1006.80	**3.91**	47.07	190.19	**0.56**	**0.00**

We highlight in bold font all Δ_*j*_ < 10. For all other models *j* there is essentially no empirical support^48^.

**Table 3:** Summary of fitted parameters of the GWB model for the investigated KKI graphs.

id	*k*_*c*_	*α*	*β*	*γ*
10	115	0.239	0.4486	110.22
11	309	1.619	0.2700	208.37
12	119	2.597	0.2382	270.62
13	35	0.669	0.3519	100.98
14	114	0.411	0.4232	199.25
15	83	0.064	0.6021	−18.51
16	101	45.670	0.0507	379.24
17	21	0.057	0.6437	−4.60
18	94	0.358	0.4260	141.72
19	112	8.329	0.1645	502.64

**Table 4:** Summary of small-world properties for the studied KKI graphs.

KKI	*N*	*N*_edges_	〈*k*〉	*L*	*L*_*r*_	*C*^*W*^	*C*^Δ^	*C*_*r*_	*σ*^*W*^	*σ*^Δ^
10	9.40 × 10^5^	8.68 × 10^7^	184.71	11.38	3.02	5.94 × 10^−1^	3.20 × 10^−1^	1.97 × 10^−4^	803.26	433.09
11	8.63 × 10^5^	7.07 × 10^7^	163.84	12.25	3.07	5.99 × 10^−1^	3.24 × 10^−1^	1.90 × 10^−4^	789.23	427.69
12	7.44 × 10^5^	4.98 × 10^7^	133.79	13.91	3.14	6.02 × 10^−1^	3.58 × 10^−1^	1.80 × 10^−4^	757.12	450.43
13	8.46 × 10^5^	5.93 × 10^7^	140.17	12.96	3.14	6.02 × 10^−1^	3.56 × 10^−1^	1.66 × 10^−4^	881.74	521.58
14	7.70 × 10^5^	5.36 × 10^7^	139.10	13.10	3.13	6.01 × 10^−1^	3.62 × 10^−1^	1.81 × 10^−4^	794.64	478.99
15	8.47 × 10^5^	6.94 × 10^7^	163.84	12.80	3.06	5.99 × 10^−1^	3.32 × 10^−1^	1.94 × 10^−4^	740.79	411.13
16	7.60 × 10^5^	5.70 × 10^7^	150.11	12.03	3.09	6.02 × 10^−1^	3.38 × 10^−1^	1.98 × 10^−4^	782.48	438.63
17	7.87 × 10^5^	5.20 × 10^7^	132.29	13.00	3.16	6.02 × 10^−1^	3.73 × 10^−1^	1.68 × 10^−4^	869.74	529.15
18	8.49 × 10^5^	6.63 × 10^7^	156.21	11.30	3.09	5.98 × 10^−1^	3.58 × 10^−1^	1.84 × 10^−4^	888.09	531.35
19	7.31 × 10^5^	4.94 × 10^7^	134.99	13.17	3.14	6.02 × 10^−1^	3.59 × 10^−1^	1.85 × 10^−4^	775.96	462.90

*N, N*_edges_: number of nodes and edges. 〈*k*〉: mean degree. *L*: average shortest path length. *L*_*r*_: expectation value for the average shortest path length in ErdŐs-Rényi graphs with the same *N* and *N*_edges_. *C*^*W*^, *C*^Δ^: clustering coefficients defined by Eqs 10 and 11, respectively. *C*_*r*_: mean clustering coefficient in ErdŐs-Rényi graphs. *σ*^*W*^, *σ*^Δ^: small-world coefficient defined by Eq. 9, based on either *C*^*W*^ or *C*^Δ^.
