# Supplementary material for: The topology of large Open Connectome networks for the human brain
Source: Sci Rep. 2016 Jun 7;6:27249. doi: 10.1038/srep27249 (PMC4895133; doi:10.1038/srep27249)
Supplement: Supplementary Information [file srep27249-s1.pdf]

Supplementary information
The topology of large Open Connectome networks for the human brain

Michael T. Gastner [1] and Géza Ódor[2]
[1] *Yale-NUS College, 16 College Avenue West, #01-220 Singapore 138527*
[2] *MTA-EK-MFA, Research Center for Energy, Hungarian Academy of Sciences, P. O. Box 49, H-1525 Budapest, Hungary*

Justification of AIC_c as model selection criterion

Among the various criteria for model selection that have been proposed in the literature, the Akaike and Bayesian information criteria (commonly abbreviated as AIC and BIC, respectively) are those that are most frequently employed in the analysis of empirical data. Both criteria have the general form

$$-2\ln(\mathcal{L}(\hat{\mathbf{v}})) + aK, \tag{S1}$$

where $\mathcal{L}$ is the likelihood function, $\hat{\mathbf{v}}$ the model parameters that maximize $\mathcal{L}$, and K is the number of parameters. The difference between AIC and BIC is the prefactor a in the last term,

$$a = \begin{cases} 2 & \text{for AIC,} \\ \ln(N) & \text{for BIC,} \end{cases} \tag{S2}$$

where N is the number of data points. For $N \geq 8$, the BIC gives thus a larger penalty than the AIC for every additional parameter in the model.

BIC-based model selection is known to be consistent in the sense that it almost surely selects the true model if it is among the candidates and N is infinitely large [1]. AIC-based model selection is generally not consistent, but unlike the BIC it is, in statistical parlance, efficient: in the limit $N \to \infty$ the AIC selects a candidate model with the smallest Kullback-Leibler divergence from the true model, even if the true model is not among the candidates [2, 3]. The true model is in most biological applications indeed unlikely to be included in the candidate set because it usually contains a large number of unknown parameters, which may furthermore increase with N. The consistency of the BIC and the efficiency of the AIC are asymptotic statements valid for $N \to \infty$. While it is insightful to know under which conditions the AIC or BIC are asymptotically optimal, these properties are ultimately of theoretical rather than practical relevance for finite data sets. The best guidance which criterion to choose comes from simulation studies involving finite data.

With a random number generator we have created finite data that pose a similar challenge to the model selection algorithm as the observed degree distributions. The data are $N = 10^6$ random numbers, independently sampled from the distribution

$$\Pr(k_{\mathrm{obs}} > k) = \begin{cases} \exp\left[-\beta(k_c+\alpha)^{-1}k\right] & \text{if } 0 < k \leq k_c, \\ C(k_c,\alpha,\beta) \times (k+\alpha)^{-\beta} & \text{if } k > k_c, \end{cases} \tag{S3}$$

where k_{obs} is the generated random integer and the prefactor on the second line is $C(k_c,\alpha,\beta) = (k_c+\alpha)^{\beta}\exp\left[-\beta(k_c+\alpha)^{-1}k_c\right]$. The tail of this distribution is a power law. If k were real-valued rather than an integer, the right-hand side of Eq. (S3) would be differentiable everywhere. We have chosen this distribution for two reasons. First, we want to test whether it is possible to determine with information criteria that the true model is a power-law (POW in Table 1). Second, we want to find out which criterion is better at determining the precise crossover point k_c in the presence of random fluctuations. The motivation is that, if the brain were indeed scale-free, we would correctly identify the power-law model and its parameterization. We fixed $\alpha = 10$, $\beta = 2.5$ and allowed k_c to vary.

For our first exploratory Monte Carlo simulations, we compared whether AIC- or BIC-based model selection is more successful at determining the true k_c from the POW candidate models

$$\Pr_{\mathrm{candidate}}(k_{\mathrm{obs}} > k) = \begin{cases} 1 - \sum_{i=1}^{k} A_i & \text{if } 0 < k \leq k_c, \\ \left(1 - \sum_{i=1}^{k_c} A_i\right)\left(\frac{k_c+\alpha}{k+\alpha}\right)^{\beta} & \text{if } k > k_c, \end{cases} . \tag{S4}$$

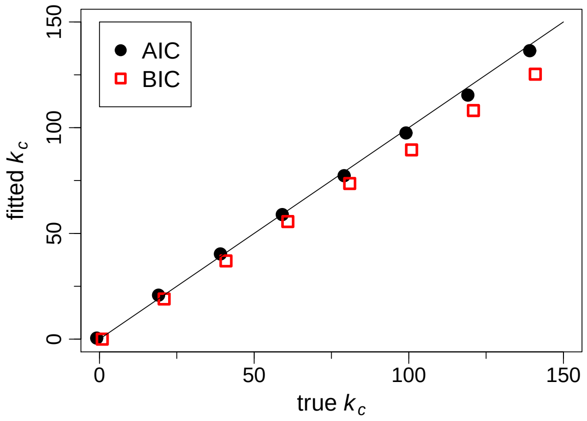

FIG. S1: The true crossover point k_c in Eq. (S3) versus values fitted by selecting among the candidate models of Eq. (S4). The selected models either minimize AIC (black filled circles) or BIC (red open squares). The diagonal line indicates perfect prediction. Standard errors are smaller than symbol sizes.

where α, β and $A_1, \ldots, A_{k_c}$ are adjustable parameters. The crossover point k_c is not itself a parameter in Eq. (S4), but rather an index for different candidate models.

We summarize the results in Fig. S1. The AIC reconstructs the crossover point almost accurately in the entire range $0 \le k_c \le 150$. The BIC underestimates k_c, especially when k_c is large. The reason lies in the BIC's steeper penalty for additional parameters in Eq. (S2). Another obstacle for the BIC is that the true model (i.e. the distribution of Eq. S3 with the three parameters α, β and k_c) is generally not one of the candidates of Eq. (S4) because those contain more parameters than necessary, in particular one parameter A_i for every $0 < i \le k_c$. The AIC, by contrast, correctly chooses a candidate that still describes the input best among the wrong (i.e. overparameterized) models: for $k > k_c$ the distribution decays $\propto (k+\alpha)^{-\beta}$ whereas there is no power law in the region $0 < k \le k_c$.

Having found that the AIC is better suited for model selection in the present context, we investigate next whether the AIC reliably identifies the correct model among those listed in Table 1. We generate 10 independent sets of $N = 10^6$ random numbers each. These are drawn from the distribution of Eq. (S3) with $\alpha = 10$, $\beta = 2.5$, and now we also fix $k_c = 100$. For 8 out of these 10 sets, the power law had the smallest AIC. In the remaining two, the power law's AIC differed from the minimum only by $\Delta_{\text{POW}} = 0.05$ in one case and $\Delta_{\text{POW}} = 0.30$ in the other case. As we have explained after Eq. (5), such small Δ_{POW} are still interpreted as substantial empirical support for the power law. We conclude that, if the connectome's degree distribution were scale-free, the AIC would have correctly identified a power law as a plausible candidate model.

We have tested whether this statement remains true even if the observed network is not the full scale-free network, but only a random sample of 20% of the edges. Such subsampling of the true network might mimic that the data are collected at a finite resolution and therefore only a fraction of the fiber tracts might be detected. For a fixed power-law degree distribution ($\alpha = 10$, $\beta = 2.5$, $N = 2 \cdot 10^6$, $k_c = 0$), we first construct a corresponding network with the configuration model [4]. We then randomly select 20% of the edges. The degrees of this subgraph have the distribution [5]

$$P(k_{\text{obs}} = j) = \sum_{i=j}^{\infty} \binom{i}{j} 0.2^j \, 0.8^{i-j} P(k_{\text{true}} = i). \tag{S5}$$

Here k_{true} is the true degree of a node (i.e. in our model a power-law distribution POW as in Table 1 of the main text) and k_{obs} is the degree observed in the sampled network. We removed all nodes with degree zero from the network to be consistent with the procedure used by the OCP.

Applying AIC-based model selection with the candidates of Table 1, we find that the differences between the heavy-tailed distributions POW, LGN, TPW and GWB are smaller for the sampled network than for the full network. In ten test runs, we could always rule out EXP, WBL and in one case LGN because $\Delta > 10$, but otherwise Δ was below this threshold. Still, POW had the smallest AIC in eight out of ten cases and came in a close second in the remaining two. In summary, AIC-based model selection would have correctly included the power law in the set of plausible candidates even if the observed network had been a small sample of a network with a scale-free degree distribution.

There is one fine detail to note when the dimension K of the model approaches the sample size N. In such cases the AIC is a negatively biased estimate of the Kullback-Leibler information [6]. An approximate correction for this bias is the additional penalty term $\frac{2K(K+1)}{N-K-1}$ in Eq. (4). This modified version of the AIC is commonly abbreviated as

AIC$_c$. Although the difference is small in the present context, it has been suggested that AIC$_c$ should routinely be used instead of the AIC [6, 7]. We have followed this advice throughout this article.

---

[1] Kim, Y., Kwon, S. & Choi, H. Consistent Model Selection Criteria on High Dimensions. *Journal of Machine Learning Research* **13**, 1037–1057 (2012).

[2] Vrieze, S. I. Model Selection and Psychological Theory: A Discussion of the Differences Between the Akaike Information Criterion (AIC) and the Bayesian Information Criterion (BIC). *Psychological Methods* **17**, 228–243 (2012).

[3] Aho, K., Derryberry, D. & Peterson, T. Model selection for ecologists: the worldviews of AIC and BIC. *Ecology* **95**, 631–636 (2014).

[4] Molloy, M. & Reed, B. A critical point for random graphs with a given degree sequence. *Random Structures & Algorithms* **6**, 161–179 (1995).

[5] Stumpf, M. P. H., Wiuf, C. & May, R. M. Subnets of scale-free networks are not scale-free: sampling properties of networks. *Proc. Nat. Acad. Sci.* **102**, 4221–4224 (2005).

[6] Hurvich, C. M. & Tsai, C.-L. Regression and time series model selection in small samples. *Biometrika* **76**, 297–307 (1989).

[7] Burnham, K. P. & Anderson, D. R. Kullback-Leibler information as a basis for strong inference in ecological studies. *Wildlife Research* **28**, 111–119 (2001).
